# A Qualitative Process Evaluation of Participant Experiences in a Feasibility Randomised Controlled Trial to Reduce Indulgent Foods and Beverages

**DOI:** 10.3390/nu15061389

**Published:** 2023-03-13

**Authors:** Claire Deborah Madigan, Andrew J. Hill, Ian Douglas Caterson, Jessica Burk, Chelsea Hendy, Anna Chalkley

**Affiliations:** 1 The Centre for Lifestyle Medicine and Behaviour (CLiMB), Loughborough University, Loughborough LE11 3TU, UK; 2 The Boden Initiative, Charles Perkins Centre, The University of Sydney, Sydney, NSW 2006, Australia; 3 Division of Psychological and Social Medicine, Leeds Institute of Health Sciences, Worsley Building, University of Leeds, Leeds LS2 9NL, UK; 4 Centre for Physically Active Learning, Western Norway University of Applied Sciences, 6856 Sogndal, Norway; 5 Wolfson Centre for Applied Research, Faculty of Health Studies, University of Bradford, Bradford BD7 1DP, UK

**Keywords:** obesity, energy-dense nutrient-poor foods, weight management

## Abstract

There is a growing interest in the effects of ultra-processed/energy-dense nutrient-poor foods on health outcomes, and few interventions to reduce their consumption have been tested. We tested a simple intervention to help people reduce the indulgences they consume (energy-dense nutrient-poor (EDNP) foods). Herein, we report the qualitative findings to understand how participants reduced their consumption by exploring intervention fidelity and the factors affecting consumption. We conducted a qualitative descriptive study of 23 adults who had taken part in a feasibility randomised controlled trial that asked participants to say no to seven indulgences/week and record what they said no to. Data were collected using face-to-face semi-structured interviews and analysed thematically. A total of 23 adults with an average BMI of 30.8 kg/m^2^ took part. Participants liked the term indulgence, as they could apply it to their normal dietary intake and make small changes. They found self-monitoring what they said no to helpful and reported that emotional eating and habits affected consumption. They had difficulty overcoming these. As most people are consuming too many foods that are EDNP, this simple intervention of “Say No” seven times/week has the potential to be developed as a public health campaign.

## 1. Introduction

In developed countries, the environment is one in which food is readily available and relatively inexpensive and there are frequent instances of food temptations and opportunities to eat [1]. This presents a challenge to people attempting to manage their weight. Food cues that signal the immediate pleasure of eating foods that are high in energy, especially those high in sugar and fat, can over-ride the desire to control eating to reach weight loss goals. One of the main predictors of weight gain is the consumption of foods that are energy-dense and high in fat and/or sugar [2,3,4]. This is because additional energy is consumed when these foods are eaten on top of daily meals [5]. Approximately 35% of diets consist of energy-dense nutrient-poor (EDNP) foods, and these are independently associated with a range of diseases [6,7,8,9]. 

There is little consensus about the characterisation of these types of foods. In Australia, they are described as discretionary foods, with the definition being “foods and drinks not necessary to provide the nutrients the body needs, but that may add variety” [10]. However, many of these are high in saturated fats, sugars, salt, and/or alcohol and are therefore described as “energy dense nutrient poor” [10]. Elsewhere in the literature, researchers/practitioners refer to these types of foods as snacks [11,12,13] or as indulgences [14]. Snacks may not always be high in fat/sugar or be energy-dense, and indulgences may mean different things to different people [10]. There is no clear consensus on what term the public understands and relates to. This constrains communication to help people change their behaviour. Herein, we refer to these foods as “indulgences”. 

There is a range of environmental drivers associated with an unhealthy diet. These include proximity to convenience shops and fast-food restaurants [15]. Frequently eating out of home is often associated with a poor diet, and many of the foods/drinks consumed can be defined as indulgences [16]. The availability of foods, and the daily “hassles” one experiences, have been associated with greater snack consumption [17]. Emotional eating in reference to negative affect and stress is associated with greater consumption of indulgences [18,19,20]. 

Appelhans and colleagues (2016) have proposed a neurobehavioural model for managing temptations in obesity treatment [21]. They suggest that there are three neurobehavioural processes that emerge when resisting temptations: reward-driven attentional bias, temporal discounting, and the cold-hot empathy gap. Indulgences are very palatable, rewarding foods, which makes them difficult to resist and may provide immediate gratification. Additionally, if people are in a hot state (i.e., if they are hungry, thirsty, or in a state of arousal), it is difficult to resist temptations. They recognised that self-regulation is important for reducing temptations, but this relies on executive function, which can be disrupted by common factors such as stress and insufficient sleep. There are many different factors affecting the consumption of indulgences, but there have been very few interventions tested outside a laboratory setting or lasting for more than a few weeks, which limits our understanding [22]. A 2016 scoping review examined interventions to reduce indulgences and was unable to identify a single discrete strategy that was helpful [22]. There were some strategies that showed potential, such as restriction/elimination and reformulation. However, most of these studies were laboratory experiments with one-off exposure opportunities. At the time of this study design, only two experiments had been completed in the field and were of a maximum of four weeks of duration [23,24]. 

Qualitative research may provide more in-depth contextual information about the factors affecting consumption. Such research has found that individuals use exercise, manipulation of the availability of tempting foods, and strategic formation of meals to manage their consumption of indulgences [25]. However, most participants had never tried to diet and were of a healthy weight, which may produce different experiences from those of people with an unhealthy weight. 

### The Say No Study 

To build on the evidence, we conducted a feasibility randomised controlled trial to examine the effectiveness of an intervention based on self-regulation theory to reduce indulgences over eight weeks [14,26]. The term indulgence was used as we thought it would be easy to understand, and it could be personalised and, therefore, perceived as more relevant, both of which have been shown to be important in changing behaviour [11]. We defined an indulgence as eating or drinking something that you enjoy but is usually thought of as “bad” or unhealthy when related to weight control. There were two intervention groups in the trial: Group 1 was asked to say no to indulgences seven times per week and write down what they said no to in a paper diary. Group 2 was asked to do the same and take a photograph of what they said no to and send the photograph to the research team for feedback. We asked participants to say no seven times per week as we wanted participants to focus on reduction rather than elimination; however, participants were free to say no more times if they so wished. On average, the intervention groups reduced the number of indulgences by 10 per week, suggesting a potentially small effect of the intervention. Other feasibility outcomes such as recruitment and follow-up rates were met. To understand if the intervention worked as intended, and to have a greater understanding of the participants’ behaviours, we conducted interviews at the end of the intervention. The aim of these interviews was to understand intervention fidelity and assess the individual meanings of what indulgences were whilst gaining a richness of data on factors affecting the consumption of indulgences. This study is important as there has been no previous qualitative research investigating individuals’ experiences in reducing indulgences as part of an intervention in a community setting. 

## 2. Methods

### 2.1. Study Design

The method of inquiry used was qualitative description [27], thereby permitting the generation of contextually rich data and allowing the diversity of experiences associated with the participants’ eating behaviours to be understood.

### 2.2. Participants and Recruitment 

Details relating to the recruitment of participants into the ‘Say No’ trial have been reported elsewhere [14]. To summarise, 45 participants were recruited, and the inclusion criteria were as follows: aged ≥18 years, with a BMI ≥ 25 kg/m^2^, and participants that were motivated to reduce the number of indulgent foods and beverages they consumed. Informed consent was obtained at the beginning of the trial, and participants were informed that they may be asked to take part in an interview about the study in their follow-up appointment at the end of the intervention (eight weeks).

Participants were recruited using convenience sampling from those participating in the trial. This meant that participants were invited to interview if they attended the follow-up. In some cases, there may have not been time to conduct an interview (due to participant or researcher availability), and, therefore, not all participants took part in an interview. We also decided to interview members of the control group to understand their experiences, and these members were invited on a first-come basis. 

This study was approved by the Royal Prince Alfred Hospital Sydney Local Health District Human Research Ethics Committee (X16-0163, HRE/16/RPAH/202) and the clinical trial registration is ACTRN12616001239459. 

### 2.3. Data Collection

A semi-structured interview schedule was developed and used as a guide when conducting the interviews to ensure that the interviews included similar content. At the beginning of the interviews, information on the researcher’s role with respect to the interviews, the aim of the interviews, the anticipated duration, and anonymity and confidentiality were provided, and the importance of the participants’ own opinions, experiences, and ideas was emphasised.

We explored the participants’ views on what an indulgence was and their experiences of saying no to indulgences. The questions examined why participants thought they were successful or not, if they struggled with trying to say no, and if they used other strategies that helped them to manage their weight. In addition, participants were asked about the process measures of the trials (i.e., sending photographs and completing questionnaires) and what improvements could be made. Probes and follow-up questions were used to provide examples and elaborate on ideas and opinions. 

All interviews took place in a quiet and private room at the Charles Perkins Centre Royal Prince Alfred Hospital Clinic (CPC-RPA), Sydney, where the ‘Say No’ trial was being conducted. All interviews were conducted by two female members of the research team (C.M. and C.H.) involved in the delivery of the trial and who had expertise in public health and clinical trials. There were no prior relationships with participants before the start of the trial. Before the interviews, participants may have previously met with the interviewer only once, depending on which researcher completed the baseline assessments for the trial. The study methods and reporting were completed in accordance with the Standards for Reporting Qualitative Research Checklist [28].

### 2.4. Data Analysis

All interviews were audio recorded and transcribed verbatim into Microsoft Word (Microsoft, Redmond, WA, USA), wherein the data were deidentified and referred to by an identification code before the transcripts were checked against the recordings for accuracy. The data analysis was led by the first author. The data were imported into NVivo (version 12.0; QSR International, Doncaster, VIC, Australia). Inductive thematic analysis was used to provide a nuanced and descriptive account of participants’ views on indulgences. In thematic analysis, the application of themes across data sets facilitates a systematic overview of the scope of the data, which allows the combination of the analysis of their meanings with their particular contexts [29].

Following Braun and Clarke’s [30] six phases of thematic analysis, the transcripts were initially reviewed in order to become familiar with the breadth and depth of content of the data and to generate preliminary ideas and notes for coding. An inductive approach to analysis was taken by segmenting the data and openly coding, whereby codes were collected under potential subcategories/subthemes or categories/themes before comparing the emerged coding clusters together and in relation to the entire data set. If new subthemes appeared from the second or third interview, the transcripts were re-read to check for any additional data falling within this subtheme. The coding was hierarchical, with variation in each theme being coded under subthemes. For example, “self-regulation of eating” had the subthemes of mindfulness, recording of consumption, behavioural goals, feedback on behaviour, and accountability. The candidate themes were subsequently revised and refined to ensure they reflected the meaning evident in the data set before being named. The analysis took a cyclical approach with several iterations made before establishing themes and subthemes emanating from the data. This iterative process of the repeated reading, reviewing, and refining of themes and subthemes while considering the whole text ensures a truthful representation of participants’ voices and experiences in qualitative descriptive studies [27].

The trustworthiness of the findings was facilitated using two methods. First, one of the co-authors (A.C.) independently checked the initial coding strategies and the coding framework generated by the first author to establish procedural reliability and conceptual credibility. Secondly, interpretations were openly discussed and appropriately challenged to achieve a final consensus by reviewing the framework and critically probing for explanations of certain decisions made by the first author.

## 3. Results

### 3.1. Participants

A total of 23 participants were recruited, and the mean age of the participants was 52 years (SD 10), and 16 (70%) of them were female. The average BMI at baseline was 30.8 (SD 3.6) kg/m^2^. Participants reported consuming 29 (SD 14) indulgences per week on average, and this reduced to 18 (SD 12) at follow-up. The weight change was −0.9 kg (SD 2.0) at follow-up. Most participants were employed or retired, and one participant was a PhD candidate. The majority were of White ethnicity, with one participant being Asian. There were five participants in the control group, eight in Group 1, and nine in Group 2. We conducted 23 interviews; the mean duration was 16.8 min (ranging from 12 to 36 min).

### 3.2. Themes Identified

**Figure 1:**
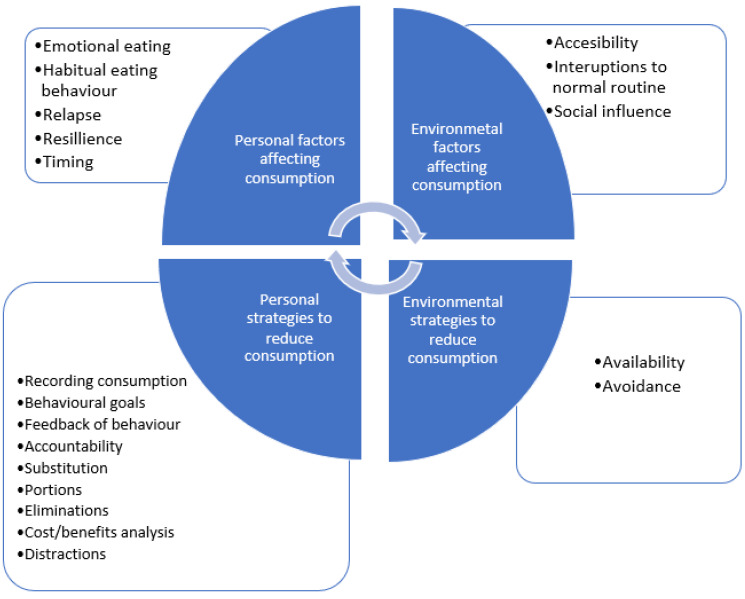
An overview of the factors affecting the consumption of indulgences and the strategies used to reduce consumption.

Five higher-order themes were identified as being important to the participants’ consumption of indulgences: the definition of an indulgence, factors affecting consumption, self-regulation, strategies individuals used to overcome consumption, and negative effects of trying to reduce consumption. Table 1 details the thematic map of the themes and subthemes identified, with additional quotes to support the findings. Figure 1 provides a visual overview of the themes and factors affecting consumption. 

### 3.3. Theme 1: The Definition of an Indulgence

The participants had specific ideas of what an indulgence was and being able to define these was important due to their individualised nature. As one participant reflected: “*I think it’s, it’s really important to allow people to determine what their own indulgence are*” (female, 55 years). Participants frequently described indulgences as specific foods or food groups. Typically, these were energy-dense and nutrient-poor. They included savoury as well as starchy foods (e.g., cheese and bread), sweet foods, and alcohol. As one participant commented:

“Cakes, chocolate. Chocolate, I didn’t go through as much. Yes, I’ve actually cut back on chocolate. But things like the pastries and the muffins and the large coffees, so. And ice creams have been on the radar a bit as well.” Male, 50 years.

Participants also referred to indulgences as an excess of food, for example, portions that were perceived to be too big or eating when not hungry. As one participant expressed:

“So, but then I was wondering about things like, so after I’ve played some tennis on the weekend, we went to somebody’s place afterwards for a kind of afternoon tea. And so having that extra slice of fruit bread with nice soft cheese on it. And really, you’re not hungry. So, I suppose that’s a good, an easy part of the definition. If you’re really not hungry and you’re just having something for the taste of it, all that.” Female, 61 years.

Participants mentioned indulgences in reference to their nutritional value, be it empty calories (i.e., no nutritional value) or what they defined as “bad foods” in relation to health:

“Usually anything processed that I know isn’t necessarily positive calories. So, anything empty calories or sugary or overly processed, I would consider an indulgence, particularly if it’s not even disguising itself as healthy food, so chocolate bars, soft drinks, things like that.” Male, 41 years.

### 3.4. Theme 2: Factors Affecting Consumption

This theme was developed to capture things participants described as influencing the consumption of indulgences. 

### 3.5. Accessibility of Food and Beverages

Participants commented on the access to and widespread availability of food and drinks. Examples included having many items available in the supermarket, access to takeaway services, and the bombardment of food advertising, which made it difficult to resist consumption.

“Well, when I go to a lot of cafes, you know, like even in the window, it’s just a carb window; I just look at it and go, “Carb, carb, carb, sugar, carb, carb, carb, sugar”. That’s it, that is the window.” Female, 55 years.

Participants also spoke about the availability of foods at supermarkets and how it affected the choices they made and the subsequent consumption of indulgences:

“I’ve got an Aldi on the corner from work and that’s been really helpful because Aldi is very limited in what they keep, but they tend to keep seasonal things … and that’s where I go, and I can buy two salmon pieces at Aldi, which are the right size for us to eat, whereas at Woolworths I’ve got to buy one and a half times what we can eat, and then it goes in the fridge and then I’ll pick at it after tea and the next morning. And I really find it hard to throw anything in the bin.” Female, 60 years.

Workplaces were frequently mentioned as a place where indulgences were available and affected consumption:

“We’re lucky enough to get food offered a few times a week and sometimes it’s pastries, sometimes it’s left-over sandwiches or salads and you know, I really know that I’ve already had my lunch; there’s no reason to want it but because it’s there and because somebody else has prepared it and of course it’s free, it’s really hard to resist.” Female, 54 years.

### 3.6. Emotional Eating

Indulgences were more likely to be consumed when feeling emotional, and this was observed as the most difficult time to try and say no. Specific examples given included when participants felt tired, sad, depressed, or stressed. During these times, the ability to resist eating indulgences often waned and participants “gave in” and consumed indulgences. This was explained by one participant who said

“Yeah, so I find, you know, I go to bed and I’m trying not to be stressed but as soon as I start thinking about planning for the rest of the week, I think that’s when it gets stressful which is when I’m more likely to eat the packet of Twisties or the ice cream and the stuff before I go to sleep.” Female, 54 years.

Another participant spoke about stressing over their weight encouraging more eating: “It’s just, I want to go down to 70 kilo at least, because now it’s over the top. And the more I stress the more I eat, and now I’m thinking, what’s going on?” Female, 61 years.

### 3.7. Influence of Others

Participants reported that social influences from friends, family, and others had both a positive and negative influence on their ability to reduce indulgences. Participants reflected that others were supportive of their attempts to change their behaviour and held them to account if they were perceived to be consuming too much.

“And it was like, normally once or twice a week, on the weekends I treat myself. I like cheese, but my daughter keeps telling me, “Mum, you’re only allowed two slices”. [laughs] “Now go away”. Or [pause], and I find it hard, and I love blue cheese.” Female, 60 years.

There were also examples shared of the positive impacts of participants influencing others to change their behaviour and engage with the intervention. As one participant explained:

“I actually got my flatmate onto it as well. I just said well we’re going to go, and this is what we’re going to do for the next eight weeks. And they lost 10.5 kilos.” Male, 33 years.

One positive influence mentioned was having young children within the house. Parents exert a level of control and responsibility over their children’s consumption, which meant that they tried to keep these foods unavailable in the home. Additionally, as the children were not consuming any indulgences, this made the adults reflect on their own behaviour:

“Really, the house is very healthy because of the young kids; they don’t get chocolate, they don’t get McDonald’s, so as long as I’m home I’m like, “Yeah, okay,” I won’t drink any beer, so I haven’t been drinking beer, so I haven’t been socialising. It’s pretty healthy. A bit boring.” Male, 42 years.

As noted above, accessibility reportedly affected consumption; however, this also interacted with social pressures or expectations to consume indulgences. One example included the challenges of social expectations:

“And it’s interesting, like, you have, kind of find yourself in certain situations where it’s expected. Like, it’ll be, I was at work the other day and there was someone’s birthday, so we’re having cakes and stuff. And I just got handed a plateful of cake. And it would have, kind of one of those situations where it would have been rude not to have, to have said, oh, actually I don’t want it, and passed it along. Whereas the, one of my colleagues had already snuck out of the room at that point. And I thought, oh, I should have just snuck out of the room and that way I wouldn’t have had to coped with it all. But yeah. So I ate it.” Female, 43 years.

### 3.8. Habitual Eating Behaviour

Participants spoke about eating behaviours being habitual in that there was a pattern of, or association with, their eating, which meant that they continued consuming indulgences. They also found it hard to change their eating behaviour.

“… What I realised it’s not so much the food, it’s just a pattern of eating, and a pattern of indulgence and pattern of, you know that you just …. It’s old habits, yeah. And it’s just choosing to ignore this, because you know, once you know that you shouldn’t be eating something, or you should be eating less of something, you can’t un-know it. You just choose to ignore it.” Female, 59 years.

### 3.9. Additional Factors Affecting Consumption

Interruptions to normal routines were reported as both positively and negatively influencing consumption. Some participants reported that while travelling, there was healthy food available and fewer indulgences, so, therefore, they changed their behaviour. This was not always the case. In other situations, there was a perception that because participants were on holiday, they could indulge more often. Some participants reported that if they had guests staying with them or special occasions, their usual eating habits changed, and this often increased their consumption of indulgences. 

Participants reported that there were specific times of days when they were likely to consume indulgences, particularly in the afternoon or evening. A few participants struggled with reducing indulgences, and we have described this as a lapse, such that participants had changed their behaviour but then started to consume the indulgences again. Some participants reported feeling that if they gave in to indulgences once during a day, they would continue to give in, seeing it as an all-or-nothing experience. A positive observation, but one that was less frequently mentioned, was resilience. This was the perceived ability to continue making changes to their consumption beyond the initial trial.

### 3.10. Theme 3: Self-Regulation

This theme was developed to describe participants’ abilities to understand and manage their own eating behaviour. Participants reported that having to record indulgences made them more conscious about what they were eating. This was described as particularly beneficial:

“I found the first part of it where we filled out the diary for a week really helpful because it made me aware exactly what I was eating. Sometimes it’s a bit hard to just keep tabs of all the snacks and you know, over at the …. School, we have our morning tea, lunch, afternoon tea but there’ll be lots of food left over from functions and stuff and so you think if you have a small square of a pastry or a cake that, you know, if you’re not writing it down, it’s easy to forget that you’ve actually had a small piece.” Female, 54 years.

As well as recording consumption, participants reported that being part of this study and having a goal helped them reduce their consumption of indulgences and, in some cases, improved their overall eating patterns. This was because it created an intention to change behaviour. Some participants also started monitoring the quantity of food and beverages they consumed, such as measuring their wine rather than freely pouring it. Participants described that recording their consumption and reflecting on their eating behaviour provided a sense of accountability for themselves and an awareness of how many indulgences they were consuming.

“Well, I’ve obviously been reasonably good, because I’ve lost nearly four kilos. It’s made me more mindful. It made me a little bit more accountable and more mindful.” Female, 54 years.

This sense of accountability extended to the researchers within the trial as participants were weighed at the end of the intervention. Many perceived this to be helpful for reducing consumption. By contrast, others spoke about needing more accountability and wanting to be weighed weekly or to have more contact with the researchers to help them reduce their food consumption:

“I was, the first weeks I was better. I was thinking more. But then I think after, as the week goes, and because I don’t have anything to report, I guess I should be feeling that, okay, accountable. Like, in a way, it’s what I need to. Like, maybe get weighed, like every week. Or once a week.” Female, 58 years.

### 3.11. Theme 4: Strategies to Reduce Consumption

This theme was developed to describe methods and techniques participants employed to reduce their consumption of indulgences. Participants explained how they reduced or could reduce the number of indulgences they consumed. The most frequently reported strategies were avoidance, accessibility, portion control, substitution, and cost/benefit analysis. Others less frequently mentioned included distraction and elimination. Participants reported avoiding indulgences by not visiting an environment where the temptation for an indulgence was more likely. For example:

“We don’t have the snacks in our rooms; we just have trays. The other rooms have a lot of snacks, so I don’t go to the other rooms.” Female, 42 years.

As noted above, the accessibility of indulgences affected consumption, but to overcome this, participants tried to make them unavailable or more difficult to access (particularly at home):

“Like, even, like, I don’t buy Magnums by the box and keep them in the fridge. And so, then I have to go to the service station, where they’re three times the price. And so you think about how much it’s going to cost you.” Female, 68 years.

Most participants reported reducing the portions they consumed by making small changes so that they consumed an indulgence, but less of it:

“But it’s like you don’t need three big pieces of toast. Just try and have one or one and a half and just try and cut back.” Female, 55 years.

Another commonly used strategy was the substitution of indulgences for foods perceived to be healthier or ‘less bad’ items.

“I’ve had a banana, like I would have a banana instead of a chocolate, or I had a cup of this fantastic tea I just discovered, instead of the sweet biscuits and a cup of tea at night, with my mum, I’ve even got my mum onto this different tea. And it’s just a herbal tea, so no milk, … but because you’ve had this herbal tea it doesn’t set up the attachment to the biscuits …” Female, 58 years.

Participants reported examples where they would weigh up the pros and cons of consuming an indulgence. That is, participants would make their decisions based on objective information regarding the costs and disadvantages of consuming the indulgence in relation to the merits, possibilities, and advantages of consuming the indulgence. This was conducted in relation to physical activity by examining how much physical activity was required in comparison with the energy equivalent of the indulgence:

“I sometimes compare that I drive my bike from my home to here; it is 25 k, it is one hour of pedalling. I just spend 700 calories, and one Tim Tam has 100 calories.” Male, 44 years.

Interestingly, one participant went further to explain how they used this strategy in combination with a reward system to help achieve another personal goal:

“Twofold things occurred, this study and also the fact that I’m now saving frantically for a deposit on a house. So, basically, any time I’ve felt like something, in the last week particularly, I’ve just gone, “No, instead of spending that and eating it, oh great I’ve said ‘No’, at the same time I’m moving the money into my savings account. So, I would literally stand in 7-Eleven, holding the bag of chips, or holding whatever I was saying “No” to, putting them back on the shelf and transferring the money straight away. So, I’ve spent the money but it’s just gone somewhere else and not in my mouth.” Female, 58 years.

A few participants reported that they decided to eliminate certain foods/drinks from their diet entirely, which meant they reduced their consumption (e.g., alcohol or not consuming butter). Another strategy was to keep busy by focusing on a specific task and avoiding thinking about the consumption of indulgences. 

### 3.12. Theme 5: Negative Effects of Trying to Reduce Consumption

This theme was used to describe unfavourable effects associated with participants’ attempts to reduce indulgences. There were a few participants who reported experiencing negative consequences of reducing indulgences, and these mainly related to the uncomfortable emotion and distress of saying no to an indulgence:

“Yes, the word “denial” is not very nice! If I deny myself something it turns up somewhere else and worse.” Female, 58 years.

Nevertheless, this participant managed to work through their belief about denial and was able to reduce their indulgences.

“And towards the end, so that was the first six weeks it was quite a battle, and then, in the last two weeks, I’ve had, something’s shifted, and I’ve actually made better choices. Even after I’ve said “No” to something really bad, I’ve then immediately chosen something good.” Female, 58 years.

Participants also felt guilty that they were not saying no to all indulgences, as one participant explained:

“Yeah, so I thought I should probably, “I’m doing this study, I shouldn’t be having that. Really, I should not be having that. The purpose of the study is not having the indulgences”, so then I was getting a bit, “No, I’m not going to be a good student for them.” Female, 58 years.

## 4. Discussion

Considering that 35% of diets consist of energy-dense nutrient-poor foods/beverages (indulgences), we need to find effective interventions to help people reduce consumption [6]. Herein, we conducted a process evaluation of a pragmatic intervention that aimed to reduce indulgences to better understand how the intervention worked and the wider factors affecting individual implementations. Participants understood the term indulgence as it allowed them to personalise the goal of saying no to their eating behaviour. Definitions varied, but included foods were those that are generally restricted when trying to manage weight. 

Participants reported that they tried to manage environmental influences on consumption by making small changes via portion control, the substitution of foods/beverages, and weighing up the pros and cons of consumption. Self-monitoring and -reflecting on the goal of trying to reduce indulgences by seven per week was reported as helpful. The results suggest it might be helpful to include strategies to break habits and reduce emotional eating in future interventions. This simple intervention of Saying No has the potential to be developed as a public health campaign. 

### 4.1. Interpretation of Findings

There are many different terms used by the public and health promotion community to define foods and beverages that individuals should have occasionally, including EDNP foods, junk food, discretionary foods, and ultra-processed foods [31]. We used “indulgences” as we thought the term would be easy to understand and personally relevant, which has been shown to be important for changing behaviour [11]. Our results support this position as participants reported varying descriptions of indulgences that were personal but would affect their weight control attempts. Some previous research asked participants in a weight loss trial to define “problem foods”. The most common problem foods were sweet baked items, salty snacks, and starchy side dishes [32]. Our findings were a little broader as participants did mention those types of foods but also spoke about consuming too much food and eating when not hungry as an indulgence. Additionally, alcohol was frequently mentioned in our interviews, and this is perhaps because beverages were not included in the other study [32]. 

There were several factors reported that increased the consumption of indulgences, including emotional eating, the influence of others, the availability of foods and beverages, and habitual eating behaviour. Our findings support the neurobehavioural model of temptations, such that if individuals are exposed to cues and are in a “hot state”, they are unlikely to be able to resist indulgences [21]. Thus, availability, the influence of others, and emotional eating appear to interact and may increase consumption. We previously found increases in daily negative affect and appetite by just one scale unit were significantly associated with an increase in the consumption of indulgences by 139 KJ per day (SE 61) and 194 KJ per day (SE 68), respectively [18]. There is a wealth of evidence suggesting that acceptance-based therapy, mindfulness, cognitive behavioural therapy, and dialectical behaviour therapy can help reduce emotional eating, but most interventions are quite intensive [33]. One such study found that those in the acceptance group lost 13.3% of their body weight at 12 months compared with 9.8% in the standard behavioural therapy group [34]. The intervention involved 25 sessions over a year and was therefore not practical to implement as a public health approach. Our intervention was very simple and brief and could be easily implemented and potentially scaled as a public health intervention. However, most participants commented on emotional eating, and, therefore, effective strategies (such as components of acceptance-based therapy or dialectical behavioural therapy [35]) that can be added may make further improvements. 

Participants often reported that eating indulgences was habitual and that it was quite hard to break the cycle of consumption. Potential strategies included the avoidance of places where indulgences were more likely to be available and/or restricting availability in the home. These strategies are similar to those found in a previous qualitative study of participants with healthy weights [25]. Participants also reported what could be described as making the habit harder to complete, or as Gardner and colleagues define it, habit inhibition, by removing the cue to eat [36]. Gardner and colleagues [36] have recently suggested that weight loss interventions have not focused on breaking habits, which could be linked to why many people regain the weight. Thus, future interventions should focus on how positive changes in behaviour can be sustained in the longer term by examining how to break habits. 

It was evident in participants’ responses that the “Say No” intervention worked primarily by promoting self-regulation. All participants reported that recording what they were consuming helped them reflect on their intake and adjust their behaviour. Self-monitoring is arguably one of the most effective techniques for changing behaviour and some participants reported that they would continue to write down what they were consuming after the intervention ended [37,38]. Participants also reported that recording what they were consuming made them accountable to themselves, as they realised what they were eating. Others wanted more external accountability, such as a weekly weigh-in. Previous research has found that in weight loss groups, being weighed weekly in front of the group leader is one of the most important factors for behavioural change [39]. We have also previously shown that accountability in self-weighing interventions results in greater weight loss compared with having no accountability [40]. Continued attendance at an intervention or follow-up creates accountability; however, this increases costs and is not sustainable in the long term. Identifying other methods of accountability is an important question that needs further research. 

Participants reported five additional strategies to help reduce indulgences: avoidance, accessibility, portion control, substitution, and weighing up the pros and cons. These have all been reported previously as helpful methods to change overall eating behaviour [22,32]. In our intervention, we focused on indulgences and not overall eating behaviour. This was based on the idea that even a 100 kcals saving of energy could prevent weight gain at the population level (participants were not told this explicitly) [41]. Consequently, most individuals spoke about making small changes to their diet, such as not consuming butter or cutting out fries. Small changes may be a sustainable method to reduce consumption of indulgences as smaller changes may be easier to maintain than larger changes. We recently conducted a systematic review of randomised controlled trials (RCTs) for weight management using a small change approach [42]. We found that there was initial evidence that it was effective for weight gain prevention, but most interventions consisted of intensive behavioural interventions. In comparison, our intervention would be easily implementable as a public health intervention [42]. 

Small changes reported by participants included reducing portion sizes. In a secondary analysis of an RCT that aimed to reduce problem foods, women who reported more frequently limiting portions of problem foods had greater weight loss at 12 months [32]. Although the long-term effects of our intervention are not known, this study provides initial support that it may be beneficial to reduce rather than eliminate indulgences. 

### 4.2. Strengths and Limitations

At the time of design, there were only two intervention studies that aimed to reduce indulgences, and no process evaluation had been completed to better understand trial effects [23,24]. Herein, the rich qualitative data provide information about how this intervention might work. Another strength of this study is that participants implemented reducing the indulgences they consumed, which gave rich data about what worked and their interpretations of what an indulgence was, rather than hypothetically reporting. Together, both the trial and this qualitative study provide evidence of some initial effects, but the results should not be overinterpreted. Our participants represent an older population and were mostly of White ethnicity, and their experiences may not reflect younger populations or other ethnic groups, who may use different strategies or respond in different ways. Selection bias could not be ruled out as participants volunteered to take part in the trial and the interviews, which may have biased the results. 

## 5. Conclusions

Given the importance of reducing the consumption of indulgences, which are independently associated with a range of health outcomes, interventions are needed to help people. Our findings suggest a simple behavioural intervention to reduce the consumption of indulgences, which worked as planned and supports the findings of the feasibility trial. Emotional and habitual eating were two areas that participants reported affecting consumption, but it was unclear how participants would manage these influences. Future research should focus on refining this intervention and testing the effectiveness of these strategies as a public health campaign.

## Figures and Tables

**Table 1:** Thematic map summarising themes generated from participant interviews.

Th	Subthemes	Example Quotations
**What is an indulgence?** A description of foods and drinks that are considered indulgences.	**Food groups** A specific food or group of foods that is considered an indulgence.	The, and probably my other indulgence, well it’s not an indulgence it’s a, it’s an addiction, is the, the dreaded sugar. Male, 67 years.
	**Excess food** Too much food is considered an indulgence.	For me, I think indulgence is when I eat too much of something, like I like eating pork, and sometimes, when I get good pork, I could eat more. And so I think if you eat something in a moderate quantity, even if it is like a chocolate or sugary thing, if you eat a little bit, that’s nothing. But if you overdo it, that’s bad. Female, 46 years.
	**Nutritional value** Related to perceived healthiness of foods.	For me, I don’t eat indulgences, so, or I don’t indulge. So what I’ve used is anything where there is possibly a healthier option to take. Male, 44 years.
**Factors affecting consumption** A factor that is reported to affect consumption but is not a specific strategy that people use to reduce consumption.	**Emotional eating** The consumption of food/drink for reasons other than hunger.	Yeah. So sometimes it would be, it was crap for me if I stay late at work. And I had, and there’s a slight emotional tag for that. So when I realize oh, blast, I’m the last one here, and so there’s a kind of a sadness with that, because I don’t have a family to race off to any more, kind of thing. And then that slightly grumpy, resentful, oh. And then if I feel hungry, as I often will, because it’s getting late. It might be 6:30, getting on to 7 o’clock, I will just think of the vending machine upstairs that has got some chips in it. Crisps, you know. And so I would often fall for that, succumb to that. Female, 61 years.
	**Interruptions to normal routine** When an interruption to perceived normal routine results in consumption changing.	It’s a little bit hard to get into a routine when my routine gets interrupted with the in-laws here for six months … ah, six weeks … Yeah. So I think those type of interruptions just doesn’t help because I can’t really do my usual walk over the bridge because I need to be home straight away so we can spend time together. I could be a little bit stricter but it’s like “No, they’re on holidays. I’m going to be on holidays.” Female, 39 years.
	**Accessibility** How easily available indulgences are.	Yeah. To an extent, I probably found it a bit harder in the domestic situation at home, especially when not much was happening. So I was having a cup and grabbing some biscuits. Instead of two I might have three or four, kind of thing. So, yeah. Male, 50 years.
	**Influence of others** How others may influence the participants’ consumption of indulgences.	Yeah, and also living with someone, it’s hard …I live with a man who rewards himself with food, who makes himself feel better, makes himself feel happy with food, and so there are times when you go, “No, I’m not going to do that”. Then other times you’re going, “Looks really nice.” Female, 59 years.
		Yeah. What’s very useful to me in that regard is that I’m very, very hands on with my grandchildren. And their parents and we are very strict with them about the clarity around what is a sometimes food and what is a, indulgence. And so, of course, if you’re imposing those standards, it makes you think a bit more about your own behaviour, which is helpful. Female, 67 years.
	**Habitual eating behaviour** Something that you do often and regularly.	I don’t know. The habitual thing is to always say, for people like me is to always say, “Yes.” Female, 58 years.
	**Relapse** An occasion when after successfully reducing indulgences, they are then consumed.	Yeah, and I don’t know, like I can go really well for weeks and weeks and weeks, and I know one little chink and then that’s it. Like this week I ate that cake; every day there was something. Like after I’d had that, normally if Alex had said, “Do you want pasta for tea tonight,” I would have said, “No,” but I thought, “Oh, I’ve had a cake and I’d like some pasta.” Female, 60 years.
	**Resilience** Continuing to reduce consumption in the face of adversity.	But I feel I’ve got a really strong resolve now and I think that’s because I really did appreciate and see the shift in, in cravings. Female, 55 years.
	**Timing** Reporting that there are specific times for consumption.	So, you know, I would like to try to eat earlier during the day because I know I’m going to be up later and continuing to lose that energy but it doesn’t work that way. I’m in such a rush in the morning, I eat very little and then as I get home and are at home, that’s when I tend to eat because I’m really hungry by then. Female, 54 years.
**Self-regulation of eating** The act of controlling one’s behaviour compared with a goal.	**Mindfulness** Consciously aware of what they are consuming.	I found I was conscious of the program and saying no and would find for the most part my diet and what I chose to eat during the day would never, I wouldn’t feel I was tempted by even having an indulgence, because I was always very happy to eat. Female, 58 years.
	**Recording consumption** Recording or measuring their eating or drinking.	But I do enjoy my wine, so … I’m just measuring it. Female, 68 years.
	**Behavioural goals** Setting behavioural goals to reduce consumption.	I just thought to myself, “No, I’m not going to have that.” So it was, but I wouldn’t have always necessarily taken it anyway; I would have just resisted it because I know it’s not good for me. But because I was doing this study, I thought about what you’re really doing, my commitment with it, and I thought, “That’s interesting, I’m going to say no to that.” Female, 58 years.
	**Feedback on behaviour** The interpretation of feedback from others or themselves via self-monitoring.	I’ll try and just have this mental check about portion size and how much I’m eating but certainly what this program has done is made me think about the indulgences and probably more the chocolate and the sweets. Female, 55 years.
	**Accountability** The perceived obligation to be answerable to someone.	I’m saying “No” because I’m in this study and I’ve got to say “No, I’m not having it.” So I’m saying “No.” They thought that was hilarious. Yes. I had a bread roll one time and, “Oh, I’d really like that.” It was at a golf lunch, yeah. No. I’m saying no to this. Oh, would you like my bread roll? Perhaps I could take a photo first. Send it over to the other side of the table …” Female, 68 years.
**Strategies people use to overcome consumption** A strategy or technique that people use to reduce the indulgences they consume.	**Substitution** Swapping an indulgence for another item that is thought of as healthier.	Other times I’ll have yoghurt and strawberries or something. So I make sure I’ve got yoghurt and strawberries. And I just have that instead of the ice cream. Female, 68 years.
	**Portions** Reducing the size or number of things that are consumed.	And when we go for coffees and things, which we do once a week, twice a week probably, we try and, we share if we have something. Like yesterday it was one muffin four ways. Female, 68 years.
	**Elimination** Removal of indulgences from their diet.	Yeah well I, they’re the same most days and I would tend to think that, you know, I have me Vita Brits or me porridge or, you know me cereal of a morning which is pretty much the same and what I used to do was put a dabble of cream on it. I: Okay and you stopped that? And I just, I wouldn’t say 100% but … most of the time. Male, 67 years.
	**Cost/benefit analysis** Weighing up the benefits and costs of consuming the indulgence.	And I thought look there’s just no nutritional value in that. It’s just a bit of comfort food. Female, 55 years.
	**Distractions** Something that prevents them from focusing on consuming indulgences.	And of course, at home it’s very hard. So what I started doing is, I keep telling myself, “Okay, I’ll eat it later.” Female, 46 years.
	**Avoidance** Purposefully avoiding a situation that would result in consuming indulgences.	And I don’t go to that section in the supermarket anymore. Male, 33 years.
	**Availability** Limiting the accessibility of indulgences.	Yeah, exactly. And that’s I think just what it said there; so we sort of made a decision at home…stop buying it, because I’ll just, I just eat it all. Male, 42 years.
**Negative consequences of saying no** **Negative affect experienced when participants say no to consuming indulgences**	**Denial** The negative feeling of not being allowed to consume an indulgence.	But it’s a lifestyle thing as well, but I don’t necessarily like not eating. I feel like I’m denying myself. I really feel like I’m denying myself all the time. Female, 60 years.

## Data Availability

Reasonable requests for access to the original transcripts can be made by contacting the corresponding author.
